# Linear Full Decoupling, Velocity Correction Method for Unsteady Thermally Coupled Incompressible Magneto-Hydrodynamic Equations

**DOI:** 10.3390/e24081159

**Published:** 2022-08-19

**Authors:** Zhe Zhang, Haiyan Su, Xinlong Feng

**Affiliations:** College of Mathematics and System Science, Xinjiang University, Urumqi 830046, China

**Keywords:** thermally coupled magneto-hydrodynamic equations, velocity correction projection algorithms, decoupling, energy stability

## Abstract

We propose and analyze an effective decoupling algorithm for unsteady thermally coupled magneto-hydrodynamic equations in this paper. The proposed method is a first-order velocity correction projection algorithms in time marching, including standard velocity correction and rotation velocity correction, which can completely decouple all variables in the model. Meanwhile, the schemes are not only linear and only need to solve a series of linear partial differential equations with constant coefficients at each time step, but also the standard velocity correction algorithm can produce the Neumann boundary condition for the pressure field, but the rotational velocity correction algorithm can produce the consistent boundary which improve the accuracy of the pressure field. Thus, improving our computational efficiency. Then, we give the energy stability of the algorithms and give a detailed proofs. The key idea to establish the stability results of the rotation velocity correction algorithm is to transform the rotation term into a telescopic symmetric form by means of the Gauge–Uzawa formula. Finally, numerical experiments show that the rotation velocity correction projection algorithm is efficient to solve the thermally coupled magneto-hydrodynamic equations.

## 1. Introduction

The unsteady incompressible MHD equation is studied in this paper. The buoyancy effect cannot be ignored in the momentum equation due to the temperature difference of fluid flow [1,2,3]. Therefore, we consider unsteady incompressible thermally coupled MHD system. This is a strongly coupled model through the famous Boussinesq approximation [1,2,4,5]. The model is a multi-physics phenomenon: first of all, the movement of the conductor under the presence of a magnetic field generates an electric current, which changes the existing electromagnetic field; then, current and magnetic field produce Lorenz forces, which accelerate fluid particles along the lines of magnetic and current; finally, incompressible MHD are usually coupled with thermal equations because of the temperature difference between the conductive current in the momentum equation and the inability to ignore the buoyancy effect. In this way, velocity, pressure, magnetic induction, and temperature in multiple physical fields are coupled. Thermally coupled incompressible MHD model has been widely used in industries and engineering such as magnetic propulsion devices, nuclear reactor technology, semiconductor manufacturing, metal hardening, casting, melting and crystal growth; see [1,3,6,7,8,9].

**Figure 1:**
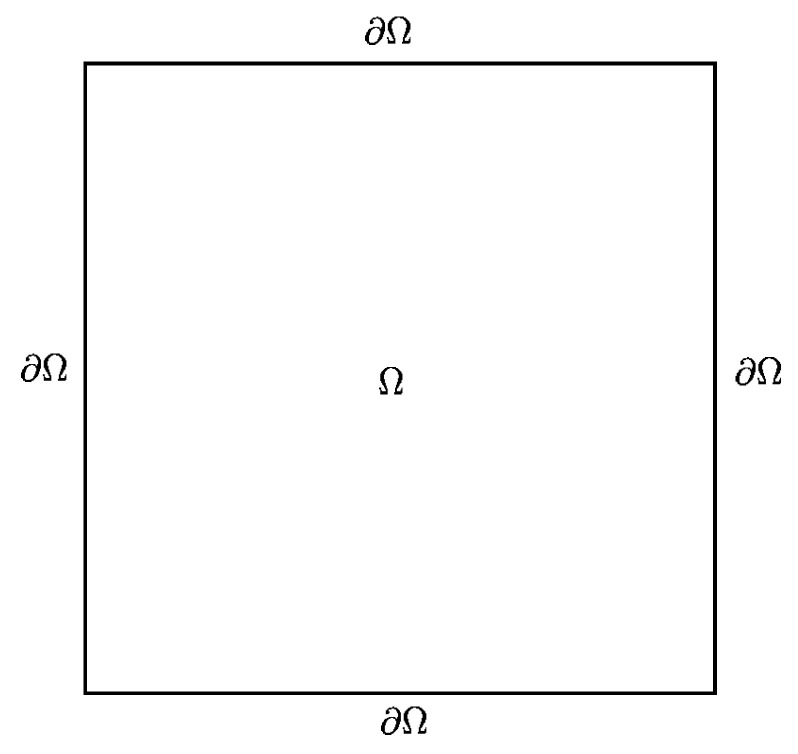
Geometry solving area.

In this paper, we consider the following unsteady thermally coupling incompressible MHD equations [1,2,4,5]:

(1) $$\left\{\begin{array}{c} \upsilon_{t}-R_{e}^{-1}\Delta \upsilon +\left(\upsilon \cdot \nabla \right)\upsilon +\nabla p-Scurlb\times b-\beta \vartheta =f,\;\mathrm{in}\;\;\Omega \times \left[0,T\right],\\ \nabla \cdot \upsilon =0,\;\mathrm{in}\;\Omega \times [0,T],\\ b_{t}+\;R_{m}^{-1}curlcurlb-curl\left(\upsilon \times b\right)=g,\;\mathrm{in}\;\Omega \times \left[0,T\right],\\ \nabla \cdot b=0,\;\mathrm{in}\;\Omega \times [0,T],\\ \vartheta_{t}-\kappa \Delta \vartheta +\upsilon \cdot \nabla \vartheta =\Psi ,\;\mathrm{in}\;\Omega \times \left[0,T\right],\\ \upsilon \left(X,0\right)=\upsilon_{0},\;b\left(X,0\right)=b_{0},\;\vartheta \left(X,0\right)=\vartheta_{0}\;\mathrm{in}\;\Omega \times \left\{0\right\},\\ \upsilon =0,\;b\times n=0,\;\vartheta =0,\;\mathrm{on}\;\partial \Omega \times 0, \end{array}\right.$$

where Ω is a bounded polygonal domain in $R^{d}\left(d=2,3\right)$ and ∂Ω is a polygon boundary, therefore, our main solution region is as shown in Figure 1, T is the final time. The functions of υ denotes the velocity field, *p* denotes the pressure, b denotes the magnetic field and ϑ denotes the temperature. f is a forcing term for the magnetic induction, g is the known applied current with ∇·g=0, Ψ is a given heat source. $\upsilon_{0}$, $b_{0}$ and $\vartheta_{0}$ are the initial velocity, the initial magnetic and the initial temperature, respectively. The initial magnetic induction $b_{0}$ satisfies $\nabla \cdot b_{0}=0$. n is the normal vector outside the unit of ∂Ω.

For considering the heat equation, as early as 1994, the existence and uniqueness of the solution of the stationary thermally coupled MHD equations were studied in [1,10,11]. In 1999, the influence of magnetic Prandt number on fluid in a three-dimensional nonlinear convection model under strong vertical magnetic field was studied in [5]. In 2010, the steady-state thermally coupled MHD equation under two gravity models was studied, and the existence and uniqueness results of corresponding weak solutions through data under certain preconditions were studied in [12]. In 2011, the use of a stable finite element method to approximate the thermally coupled MHD problem was proposed, and a numerical formula to solve this equation was proposed in [2]. In 2018, the MHD equation from the heat equation at each time step by using a partitioning method was decoupled, refer to [3]. Recently, the convergence analysis of the thermally coupled MHD Crank-Nicolson extrapolation full-discrete scheme was proposed in [4]. A linearized projection scheme for non-stationary incompressible coupled the MHD with heat equations is introduced; meanwhile, a linearized fully discrete scheme is proposed in [13]. Although considerable work has been conducted to develop efficient schemes for both steady and unsteady thermally coupled MHD model. Little attention has been paid to the fully decoupled scheme of unsteady thermally coupled MHD model.

In order to design an efficient and stable numerical scheme for the thermally coupled MHD equations, the main difficulties we will face are: (i) strong nonlinear terms in the MHD equations and heat equation; (ii) velocity and temperature are coupled due to buoyancy effects; due to Lorentz force and Ohm’s law, the velocity field is coupled with the magnetic field; (iii) due to the incompressible condition of velocity; namely, ∇·υ=0, the velocity and pressure are strongly coupled together, forming a saddle point problem; (iv) due to the artificial Neumann boundary condition of pressure, the numerical boundary layer will be generated, so that the $L^{2}$-norm of pressure and the $H^{1}$-norm of velocity cannot reach the optimal order; (v) due to the incompressible condition of the magnetic field; namely, ∇·b=0, this cause the model to produce singular solutions. Therefore, it is necessary to design a linear, fully decoupled and stable format for the model. To deal with (iii) difficulty encountered in the model, a common strategy to decouple the computation of the pressure from the velocity is a projection-type schemes as in the case for Navier–Stokes equations, refer to [14,15].

Projection methods can be viewed as fractional/splitting step methods, where convection diffusion and incompressibility are dealt with in two steps, refer to [14,15,16,17,18,19]. For velocity correction projection methods, sticky item is displayed processed or ignored. The pressure is made explicit in the first sub-step and is corrected in the second one by projecting the provisional velocity onto the space of incompressible vector fields. The velocity obtained in the convection-diffusion sub-step is projected in order to satisfy the weak incompressibility condition. It’s well known that SVC projection methods which the projection step precedes the viscous step, one could also refer to these methods as “projection-diffusion” methods as in [20]. This method creates artificial Neumann boundary conditions for pressure which cuts down the accuracy of the pressure approximation. The RVC scheme is proposed in [18,21,22], that leads to improved pressure approximation. More importantly and appealing, using projection methods only solves a sequence of decoupled elliptic equations for the velocity and the pressure at each time step; meanwhile, it is very efficient for large scale numerical simulations. In order to decouple the velocity and pressure, we choose the SVC and RVC in the velocity correction projection methods. The RVC scheme not only solves the problem of velocity and pressure coupling in the model, but also overcomes the limitation of artificial Neumann boundary condition for the pressure, thus improving the error accuracy of pressure.

To overcome the above difficulties, we use first-order scheme marching in time of RVC projection method to solve time dependent thermally coupled MHD problem. We deal with difficulties (i) and (ii) by using implicit-explicit format processing for nonlinear terms refer to [2,4,23], so we can decouple velocity, magnetic, temperature from the model. In addition, the velocity correction projection formats are used to solve difficulties (iii)–(iv). In order to overcome the difficulty (v), H(curl,Ω) is selected for the magnetic field and H(curl)-conforming N$\overset{´}{e}$d$\overset{´}{e}$lect element approximation is used for the magnetic field selection, thus the singularity of solutions in non-convex regions is avoided. Therefore, the difficulties (i)–(v) are successfully solved. After the above processing, the complex nonlinear saddle-point system is transformed into a series of simple linear elliptic problems, which greatly improves the computational efficiency. For space approximation, we use uniform finite elements: $P_{1}^{b}$ to discrete υ and ϑ, the b is implemented by H(curl)-conforming $N\overset{´}{e}d\overset{´}{e}\mathrm{lec}$ element, the continuous $P_{1}$ finite element for discretizing *p*. Besides, we provide the energy stability for the velocity correction projection methods. Last, numerical experiments verify the stability and convergence of the RVC algorithm.

The rest of this paper is organized as follow. In Section 2, we propose some notations for the magneto-thermal coupling model. In Section 3, we put forward a linear full decoupling velocity correction algorithms for system (1). Meanwhile, we give the corresponding stability of the proposed algorithms and derive a detailed proofs. In Section 4, to further verify the stability and effectiveness of the considered model and RVC algorithm we conduct corresponding numerical experiments, and fully demonstrate the advantages of the RVC algorithm. Finally, we summarize the conclusions of the article and make an outlook for future research work in Section 5.

## 2. Functional Setting for the Magneto-Thermal Coupling Model

To begin with, the following spaces are defined by:

$$\begin{array}{c} X:=H_{0}^{1}\left(\Omega \right)=\left\{\upsilon \in H^{1}\left(\Omega \right):\upsilon {|}_{\partial \Omega }=0\right\},\\ K:=H\left(\mathrm{curl},\Omega \right)=\{s\in L^{2}\left(\Omega \right):\nabla \times s\in L^{2}\left(\Omega \right)\},\\ W:=H_{0}\left(\mathrm{curl},\Omega \right)=\left\{s\in L^{2}\left(\Omega \right):\nabla \times s\in L^{2}\left(\Omega \right),n\times s{|}_{\partial \Omega }=0\right\},\\ M:=\{s\in W:\nabla \cdot s=0\},\\ G:=L_{0}^{2}\left(\Omega \right)=\left\{q\in L^{2}\left(\Omega \right):\int_{\Omega }qdx=0\right\},\\ Y:=H^{1}\left(\Omega \right),Y_{0}=\;{\theta \in Y,\theta |}_{\partial \Omega }=0\;. \end{array}$$

In references [24,25], we know the following Poincar$\overset{´}{e}$ inequalities and embedded inequality for *C* is a generic coefficient, where the space $L^{2}\left(\Omega \right)$ is equipped with the inner product (·,·) and $L^{2}$-norm ∥·∥.

$$\begin{array}{c} ∥u∥\leq C∥\nabla u∥,\;u\in X,\\ ∥\nabla u∥\leq C∥\nabla \cdot u∥+C∥\nabla \times u∥,\;u\in X,\\ {∥c∥}_{H(\mathrm{curl},\Omega )}^{2}={∥c∥}^{2}+{∥\nabla \times c∥}^{2},\;c\in W. \end{array}$$

Definition and properties of the nolinear form b(·,·,·), for all u,r,υ∈X are represented as follows:

$$\begin{array}{c} b(\upsilon ,u,r)=(\upsilon \cdot \nabla u,r),\\ b(\upsilon ,u,r)=-b(\upsilon ,r,u),\\ b(r,\upsilon ,\upsilon )=0, \end{array}$$

and we achieve:

$${∥\nabla \times u∥}^{2}{+∥\nabla \cdot u∥}^{2}={∥\nabla u∥}^{2},\;\;\forall u\in X.$$

**Lemma** **1.** *For any (υ,p,b,ϑ) that satisfy scheme (1), the system energy of the model (1), defined by (cf. [4]),*

(2) $$\begin{array}{c} \int_{0}^{t}(R_{e}^{-1}{∥\nabla \upsilon ∥}^{2}+R_{m}^{-1}{∥\nabla \times b∥}^{2}+{\kappa ∥\nabla \vartheta ∥}^{2})dt+\frac{1}{2}{∥\upsilon \left(t\right)∥}^{2}+\frac{1}{2}{∥b\left(t\right)∥}^{2}+\frac{1}{2}{∥\vartheta \left(t\right)∥}^{2}\\ =\frac{1}{2}{∥\upsilon \left(0\right)∥}^{2}+\frac{1}{2}{∥b\left(0\right)∥}^{2}+\frac{1}{2}{∥\vartheta \left(0\right)∥}^{2}+\int_{0}^{t}\left(\left(f+\beta \theta ,\upsilon \right)+\left(g,b\right)+\left(\Psi ,\vartheta \right)\right)dt. \end{array}$$

**Lemma** **2** (The inner product identity of the time derivative term). *Let us call δ the difference quotient of two continuous functions. For example, for any sequence of functions ${\left\{v^{k}\right\}}_{k=0}^{N}$, N is the $\left[\frac{T}{▵t}\right]$, make (cf. [26])*

$$\begin{array}{c} \delta v^{k+1}=v^{k+1}-v^{k},\delta \delta v^{k+1}=\delta \left(\delta v^{k+1}\right)=v^{k+1}-2v^{k}+v^{k-1},\cdots \cdots \end{array}$$

*have*

$$\begin{array}{c} 2\left(v^{k-1}-v^{k},v^{k+1}\right)=∥v^{k+1}{∥}^{2}-∥v^{k}{∥}^{2}+{∥v^{k+1}-v^{k}∥}^{2}. \end{array}$$

## 3. Linear Full Decoupling Algorithms and Their Stabilities

In the section, firstly, we present linear fully decoupling SVC scheme and stability for thermally coupled incompressible MHD equations. However, SVC scheme can cause obviously an artificial Neumann boundary condition for the pressure, which cuts down the accuracy of the pressure approximation. Therefore, in order to deal with the limitation of artificial Neumann boundary conditions for the pressure, we propose an RVC scheme for this model and give its stability.

We set $0\leq k\leq [\frac{T}{▵t}]$ where the *T* stands termination time and ▵t>0 expresses the time step size. Initial conditions $(\upsilon^{0}$, $b^{0}$, $\vartheta^{0}$, $p^{0})$ are given, we compute $({\tilde{\upsilon }}^{k+1}$, $b^{k+1}$, $\vartheta^{k+1}$, $p^{k+1})$.

**Remark** **1.** *In fact, the solution for the magnetic introduction still satisfies the weakly divergence free property. Since ∇φ∈W for all $\varphi \in H_{0}^{1}\left(\Omega \right)$, by choosing C=∇φ, we can obtain $(b_{t},\nabla \varphi )=0$; namely, $(\frac{b^{k+1}-b^{k}}{\Delta t},\nabla \varphi )=0$. Due to $(b^{0},\nabla \varphi )=0$, it implies that $(b^{k+1},\nabla \varphi )=0$, where k=0,1,⋯,N. Thus, there is no need to add a Lagrange multiplier in the magnetic equation as in [27].*

**Remark** **2.** *The SVC scheme’s boundary condition for the pressure filed is artificial Neumann boundary [15,20]. From the Equation (6), we get $\left(\upsilon^{k+1}\cdot \nabla {\tilde{\upsilon }}^{k+1}-R_{e}^{-1}\Delta {\tilde{\upsilon }}^{k+1}\right)\cdot n{{|}_{\partial \Omega }=\left(\upsilon^{k}\cdot \nabla {\tilde{\upsilon }}^{k}-R_{e}^{-1}\Delta {\tilde{\upsilon }}^{k}\right)\cdot n|}_{\partial \Omega }$, then combine with (5), pressure field boundary condition is obtained*

$$\begin{array}{c} \nabla p^{k+1}\cdot n{{|}_{\partial \Omega }=\left(f^{k+1}+\beta \vartheta^{k+1}+S\nabla \times b^{k+1}\times b^{k}-\upsilon^{0}\cdot \nabla {\tilde{\upsilon }}^{0}+R_{e}^{-1}\Delta {\tilde{\upsilon }}^{0}\right)\cdot n|}_{\partial \Omega }. \end{array}$$

**Remark** **3.** *In a bid to prove the SVC scheme’s stability, we need to define*

$$\begin{array}{c} \omega^{k}=-R_{e}^{-1}\Delta {\tilde{\upsilon }}^{k}+\upsilon^{k}\cdot \nabla {\tilde{\upsilon }}^{k}. \end{array}$$

**Theorem** **1** (SVC scheme’s stability). *For all 0≤k≤N, for any (υ,p,b,ϑ) that satisfy the Algorithm 1 is stable in the sense that*

$$\begin{array}{c} S∥b^{N}{∥}^{2}+∥\vartheta^{N}{∥}^{2}+∥{\tilde{\upsilon }}^{N}{∥}^{2}+\Delta t^{2}∥\omega^{N}{∥}^{2}+2\Delta t\sum_{k=1}^{N}(\kappa ∥\nabla \vartheta^{k}{∥}^{2}+SR_{m}^{-1}{∥\nabla \times b^{k}∥}^{2}\\ +R_{e}^{-1}∥\nabla {\tilde{\upsilon }}^{k}{∥}^{2})\leq S∥b^{0}{∥}^{2}+∥{\tilde{\upsilon }}^{0}{∥}^{2}+∥\vartheta^{0}{∥}^{2}+\Delta t^{2}∥\omega^{0}{∥}^{2}+2\Delta t\sum_{k=1}^{N}\left((f^{k}+\beta \vartheta^{k},\upsilon^{k}\right)\\ +S\left(g^{k},b^{k}\right)+\left(\Psi^{k},\vartheta^{k}\right)). \end{array}$$

**Algorithm 1** SVC SchemeStep 1. Find $\upsilon_{☆}^{k+1}\in X,b^{k+1}\in W$ such that:

(3)
$$\left\{\begin{array}{c} \frac{b^{k+1}-b^{k}}{▵t}+R_{m}^{-1}\nabla \times \left(\nabla \times b^{k+1}\right)-\nabla \times \left(\upsilon_{☆}^{k}\times B^{k}\right)=g^{k+1},\\ \frac{\upsilon_{☆}^{k}-{\tilde{\upsilon }}^{k}}{▵t}-S\nabla \times b^{k+1}\times b^{k}=0,\\ b^{k+1}{\times n|}_{\partial \Omega }=0. \end{array}\right.$$

Step 2. Solve $\vartheta^{k+1}\in Y_{0}$ such that:

(4)
$$\left\{\begin{array}{c} \frac{\vartheta^{k+1}-\vartheta^{k}}{▵t}-\kappa \Delta \vartheta^{k+1}+{\tilde{\upsilon }}^{k}\cdot \nabla \vartheta^{k+1}=\Psi^{k+1},\\ \vartheta^{k+1}{|}_{\partial \Omega }=0. \end{array}\right.$$

Step 3. Solve $\upsilon^{k+1}\in X,p^{k+1}\in Q$ such that:

(5)
$$\left\{\begin{array}{c} \frac{\upsilon^{k+1}-\upsilon_{☆}^{k}}{▵t}-R_{e}^{-1}\Delta {\tilde{\upsilon }}^{k}+\upsilon^{k}\cdot \nabla {\tilde{\upsilon }}^{k}+\nabla p^{k+1}=f^{k+1}+\beta \vartheta^{k+1},\\ \nabla \cdot \upsilon^{k+1}=0,\;\;\;\upsilon^{k+1}{\cdot n|}_{\partial \Omega }=0. \end{array}\right.$$

Step 4. Update $\upsilon^{k+1}\in X$ by working out ${\tilde{\upsilon }}^{k+1}\in X$:

(6)
$$\left\{\begin{array}{c} \frac{{\tilde{\upsilon }}^{k+1}-\upsilon^{k+1}}{▵t}-R_{e}^{-1}\Delta \left({\tilde{\upsilon }}^{k+1}-{\tilde{\upsilon }}^{k}\right)+\upsilon^{k+1}\cdot \nabla {\tilde{\upsilon }}^{k+1}-\upsilon^{k}\cdot \nabla {\tilde{\upsilon }}^{k}=0,\\ {\tilde{\upsilon }}^{k+1}{|}_{\partial \Omega }=0. \end{array}\right.$$

**Proof.**  Taking inner product from both sides for the first equation of (3) with $2\Delta tSb^{k+1}$, we have

(7) $$\begin{array}{c} S∥b^{k+1}{∥}^{2}-S∥b^{k}{∥}^{2}+S∥b^{k}-b^{k+1}{∥}^{2}+2\Delta tSR_{m}^{-1}{∥\nabla \times b^{k+1}∥}^{2}\\ -2\Delta tS\left(\upsilon_{☆}^{k}\times b^{k},\nabla \times b^{k+1}\right)=2\Delta tS\left(g^{k+1},b^{k+1}\right), \end{array}$$

the second equation of (3), taking inner product with $2\Delta t\upsilon_{☆}^{k}$, we find

(8) $$∥\upsilon_{☆}^{k}{∥}^{2}-∥{\tilde{\upsilon }}^{k}{∥}^{2}+{∥\upsilon_{☆}^{k}-{\tilde{\upsilon }}^{k}∥}^{2}+2\Delta tS\left(\upsilon_{☆}^{k}\times b^{k},\nabla \times b^{k+1}\right)=0.$$

For the first equation of (4), taking inner product with $2\Delta t\vartheta^{k+1}$, using Lemma 2 and the property (υ·∇s,s)=0, we achieve

(9) $$∥\vartheta^{k+1}{∥}^{2}-∥\vartheta^{k}{∥}^{2}+∥\vartheta^{k+1}-\vartheta^{k}{∥}^{2}+2\Delta t\kappa {∥\nabla \vartheta^{k+1}∥}^{2}=2\Delta t\left(\Psi^{k+1},\vartheta^{k+1}\right).$$

Using $\omega^{k}$ and taking inner product with $2\Delta t\upsilon^{k+1}$, we obtain

(10) $$\begin{array}{cc} ∥\upsilon^{k+1}{∥}^{2}-∥\upsilon_{☆}^{k}{∥}^{2}+{∥\upsilon^{k+1}-\upsilon_{☆}^{k}∥}^{2}\; & +2\Delta t\left(\omega^{k},\upsilon^{k+1}\right)=2\Delta t\left(f^{k+1}+\beta \vartheta^{k+1},\upsilon^{k+1}\right), \end{array}$$

on the other hand, we rearrange (6) as

(11) $${\tilde{\upsilon }}^{k+1}+\Delta t\omega^{k+1}=\upsilon^{k+1}+\Delta t\omega^{k},$$

we recall that if b(υ,υ,v)=0, then $\left(\omega^{k+1},\upsilon^{k+1}\right)=R_{e}^{-1}{∥\nabla \upsilon^{k+1}∥}^{2}$, taking inner product with itself for the first equation of (11), we get

(12) $$\begin{array}{cc} ∥{\tilde{\upsilon }}^{k+1}{∥}^{2}-∥\upsilon^{k+1}{∥}^{2}+\Delta t^{2}(∥\omega^{k+1}{∥}^{2}-∥\omega^{k}{∥}^{2})\; & +2R_{e}^{-1}\Delta t{∥\nabla {\tilde{\upsilon }}^{k+1}∥}^{2}\\ \; & +2\Delta t(\omega^{k},\upsilon^{k+1})=0, \end{array}$$

summing up (7)–(10) and (12) from k=0 to N−1 and getting rid of some positive terms, there holds

(13) $$\begin{array}{cc} \; & S∥b^{N}{∥}^{2}+∥\vartheta^{N}{∥}^{2}+∥{\tilde{\upsilon }}^{N}{∥}^{2}+\Delta t^{2}∥\omega^{N}{∥}^{2}+2\Delta t\sum_{k=1}^{N}(\kappa ∥\nabla \vartheta^{k}{∥}^{2}+SR_{m}^{-1}{∥\nabla \times b^{k}∥}^{2}\\ \; & +R_{e}^{-1}∥\nabla {\tilde{\upsilon }}^{k}{∥}^{2})\leq S∥b^{0}{∥}^{2}+∥{\tilde{\upsilon }}^{0}{∥}^{2}+∥\vartheta^{0}{∥}^{2}+\Delta t^{2}∥\omega^{0}{∥}^{2}+2\Delta t\sum_{k=1}^{N}(\left(f^{k}+\beta \vartheta^{k},\upsilon^{k}\right)\\ \; & +S\left(g^{k},b^{k}\right)+\left(\Psi^{k},\vartheta^{k}\right)). \end{array}$$

thus, the proof is ended. □

**Remark** **4.** *The RVC scheme’s boundary condition for the pressure filed is consistent boundary [21]. From the Equation (18), we get $\left(\upsilon^{k+1}\cdot \nabla {\tilde{\upsilon }}^{k+1}-R_{e}^{-1}\Delta {\tilde{\upsilon }}^{k+1}\right)\cdot n{{|}_{\partial \Omega }=\left(\upsilon^{k}\cdot \nabla {\tilde{\upsilon }}^{k}+R_{e}^{-1}\nabla \times \left(\nabla \times {\tilde{\upsilon }}^{k}\right)\right)\cdot n|}_{\partial \Omega }$, then combine with (17), pressure field boundary condition is obtained*

$$\begin{array}{c} \nabla p^{k+1}\cdot n{{|}_{\partial \Omega }=\left(f^{k+1}+\beta \vartheta^{k+1}-\upsilon^{k+1}\cdot \nabla {\tilde{\upsilon }}^{k+1}+R_{e}^{-1}\Delta {\tilde{\upsilon }}^{k+1}+S\nabla \times b^{k+1}\times b^{k}\right)\cdot n|}_{\partial \Omega }. \end{array}$$

**Remark** **5.** *To decouple the magnetic field b and the velocity field υ, the velocity $\upsilon_{☆}^{k}$ can be computed directly from the Equation (15).*

$$\upsilon_{☆}^{k}={\tilde{\upsilon }}^{k}+▵tS\nabla \times b^{k+1}\times b^{k},$$

*we get a linear equation as follows*

$$\frac{b^{k+1}-b^{k}}{▵t}+R_{m}^{-1}\nabla \times \left(\nabla \times b^{k+1}\right)-\nabla \times (\left({\tilde{\upsilon }}^{k}+▵tS\nabla \times b^{k+1}\times b^{k}\right)\times B^{k})=g^{k+1},$$

*thus, we can calculate $b^{k+1}$, by the aid of $b^{k+1}{\times n|}_{\partial \Omega }=0$.*

**Remark** **6.** *Since ${\tilde{\upsilon }}^{k}$ is known, we can directly calculate the temperature in (16) and take it into (17) to apply directly. We use a special technique to obtain $p^{k+1}$ from the third equation of (17) and Equation (18). Using (17)${}^{k+1}$− (17)${}^{k}$− (18)${}^{k}$, we obtain*

$$\begin{array}{cc} \; & \frac{\upsilon^{k+1}-2{\tilde{\upsilon }}^{k}-{\tilde{\upsilon }}^{k-1}}{\Delta t}+R_{e}^{-1}\nabla \times \nabla \times {\tilde{\upsilon }}^{k}+R_{e}^{-1}\Delta {\tilde{\upsilon }}^{k}+\nabla \left(p^{k+1}-p^{k}\right)\\ \; & -\beta \left(\vartheta^{k+1}-\vartheta^{k}\right)-S\nabla \times b^{k+1}\times b^{k}-S\nabla \times b^{k}\times b^{k-1}=f^{k+1}-f^{k}, \end{array}$$

*taking divergence for the above equation with ∇·(∇×υ)=0 and $\nabla \cdot \upsilon^{k+1}=0$, we can get*

$$\frac{\nabla \cdot (-2{\tilde{\upsilon }}^{k}+{\tilde{\upsilon }}^{k-1})}{\Delta t}+\Delta \left(p^{k+1}-p^{k}+R_{e}^{-1}\nabla \cdot {\tilde{\upsilon }}^{k}\right)-\beta \nabla \cdot \left(\vartheta^{k+1}-\vartheta^{k}\right)=\nabla \cdot \left(f^{k+1}-f^{k}\right),$$

*it is worth noting that we can calculate ${\tilde{\upsilon }}^{k-1}$ with the initial value and backward Euler scheme.*

**Remark** **7.** *Finally, we update ${\tilde{\upsilon }}^{k+1}$ from the original equation with the boundary condition*

$$\left\{\begin{array}{c} \frac{{\tilde{\upsilon }}^{k+1}-{\tilde{\upsilon }}^{k}}{▵t}-R_{e}^{-1}\Delta {\tilde{\upsilon }}^{k+1}+\upsilon^{k+1}\cdot \nabla {\tilde{\upsilon }}^{k+1}+\nabla p^{k+1}+\upsilon^{k+1}-\beta \vartheta^{k+1}-S\nabla \times b^{k+1}\times b^{k}=f^{k+1},\\ {\tilde{\upsilon }}^{k+1}{|}_{\partial \Omega }=0, \end{array}\right.$$

*then, all the unknowns variables υ, b, ϑ, p are fully calculating.*

**Remark** **8.** *In a bid to prove the stability, we use the Gauge–Uzawa format. Then, we recommend a Gauge variable $\xi^{k}$ and an instrumental variable $\alpha^{k}$, namely*

$$\left\{\begin{array}{c} \xi^{0}=0,\xi^{k+1}=\nabla \cdot {\tilde{\upsilon }}^{k+1}+\xi^{k},k\geq 0,\\ \alpha^{k}=R_{e}^{-1}\nabla \times \left(\nabla \times {\tilde{\upsilon }}^{k}\right)+\upsilon^{k}\cdot \nabla {\tilde{\upsilon }}^{k}-R_{e}^{-1}\nabla \xi^{k}, \end{array}\right.$$

*so (18) becomes*

(14) $$\left\{\begin{array}{c} {\tilde{\upsilon }}^{k+1}+\Delta t\alpha^{k+1}=\upsilon^{k+1}+\Delta t\alpha^{k},\\ {\tilde{\upsilon }}^{k+1}{|}_{\partial \Omega }=0. \end{array}\right.$$

**Theorem** **2.** *$\left(RVC\;scheme^{\prime }s\;stability\right)$ For all 0≤k≤N, for any (υ,p,b,ϑ) that satisfy the Algorithm 2 is stable in the sense that*

$$\begin{array}{cc} \; & S∥b^{N}{∥}^{2}+∥\vartheta^{N}{∥}^{2}+∥{\tilde{\upsilon }}^{N}{∥}^{2}+\Delta t^{2}∥\alpha^{N}{∥}^{2}+R_{e}^{-1}\Delta t∥\xi^{N}{∥}^{2}+2\Delta t\sum_{k=1}^{N}(\kappa ∥\nabla \vartheta^{k}{∥}^{2}\\ \; & +SR_{m}^{-1}∥\nabla \times b^{k}{∥}^{2})+R_{e}^{-1}\Delta t\sum_{k=1}^{N}(∥\nabla {\tilde{\upsilon }}^{k}{∥}^{2}+∥\nabla \times {\tilde{\upsilon }}^{k}{∥}^{2})\leq S∥b^{0}{∥}^{2}+{∥\vartheta^{0}∥}^{2}\\ \; & +∥{\tilde{\upsilon }}^{0}{∥}^{2}+\Delta t^{2}∥\alpha^{0}{∥}^{2}+R_{e}^{-1}\Delta t{∥\xi^{0}∥}^{2}+2\Delta t\sum_{k=1}^{N}\left(\left(f^{k}+\beta \vartheta^{k},\upsilon^{k}\right)+S\left(g^{k},b^{k}\right)+\left(\Psi^{k},\vartheta^{k}\right)\right). \end{array}$$

**Algorithm 2** RVC SchemeStep 1. Find $\upsilon_{☆}^{k+1}\in X,b^{k+1}\in W$ such that:

(15)
$$\left\{\begin{array}{c} \frac{b^{k+1}-b^{k}}{▵t}+R_{m}^{-1}\nabla \times \left(\nabla \times b^{k+1}\right)-\nabla \times \left(\upsilon_{☆}^{k}\times B^{k}\right)=g^{k+1},\\ \frac{\upsilon_{☆}^{k}-{\tilde{\upsilon }}^{k}}{▵t}-S\nabla \times b^{k+1}\times b^{k}=0,\\ b^{k+1}{\times n|}_{\partial \Omega }=0. \end{array}\right.$$

Step 2. Solve $\vartheta^{k+1}\in Y_{0}$ such that:

(16)
$$\left\{\begin{array}{c} \frac{\vartheta^{k+1}-\vartheta^{k}}{▵t}-\kappa \Delta \vartheta^{k+1}+{\tilde{\upsilon }}^{k}\cdot \nabla \vartheta^{k+1}=\Psi^{k+1},\\ \vartheta^{k+1}{|}_{\partial \Omega }=0. \end{array}\right.$$

Step 3. Solve $\upsilon^{k+1}\in X,p^{k+1}\in Q$ such that:

(17)
$$\left\{\begin{array}{c} \frac{\upsilon^{k+1}-\upsilon_{☆}^{k}}{▵t}+\upsilon^{k}\cdot \nabla {\tilde{\upsilon }}^{k}+R_{e}^{-1}\nabla \times \nabla \times {\tilde{\upsilon }}^{k}+\nabla p^{k+1}=f^{k+1}+\beta \vartheta^{k+1},\\ \nabla \cdot \upsilon^{k+1}=0,\;\;\;\upsilon^{k+1}{\cdot n|}_{\partial \Omega }=0. \end{array}\right.$$

Step 4. Update $\upsilon^{k+1}\in X$ by working out ${\tilde{\upsilon }}^{k+1}\in X$:

(18)
$$\left\{\begin{array}{c} \frac{{\tilde{\upsilon }}^{k+1}-\upsilon^{k+1}}{▵t}-R_{e}^{-1}\Delta {\tilde{\upsilon }}^{k+1}+\upsilon^{k+1}\cdot \nabla {\tilde{\upsilon }}^{k+1}-\upsilon^{k}\cdot \nabla {\tilde{\upsilon }}^{k}-R_{e}^{-1}\nabla \times \nabla \times {\tilde{\upsilon }}^{k}=0,\\ {\tilde{\upsilon }}^{k+1}{|}_{\partial \Omega }=0. \end{array}\right.$$

**Proof.**  Taking inner product from both sides for the first equation of (15) with $2\Delta tSb^{k+1}$, we have

(19) $$\begin{array}{cc} \; & S∥b^{k+1}{∥}^{2}-S∥b^{k}{∥}^{2}+S∥b^{k}-b^{k+1}{∥}^{2}+2\Delta tSR_{m}^{-1}{∥\nabla \times b^{k+1}∥}^{2}\\ \; & -2\Delta tS\left(\upsilon_{☆}^{k}\times b^{k},\nabla \times b^{k+1}\right)=2\Delta tS\left(g^{k+1},b^{k+1}\right), \end{array}$$

the second equation of (15), taking inner product with $2\Delta t\upsilon_{☆}^{k}$, we find

(20) $$∥\upsilon_{☆}^{k}{∥}^{2}-∥{\tilde{\upsilon }}^{k}{∥}^{2}+{∥\upsilon_{☆}^{k}-{\tilde{\upsilon }}^{k}∥}^{2}+2\Delta tS\left(\upsilon_{☆}^{k}\times b^{k},\nabla \times b^{k+1}\right)=0.$$

For the first equation of (16), taking inner product with $2\Delta t\vartheta^{k+1}$, using Lemma 2 and the property (υ·∇s,s)=0, we achieve

(21) $$∥\vartheta^{k+1}{∥}^{2}-∥\vartheta^{k}{∥}^{2}+∥\vartheta^{k+1}-\vartheta^{k}{∥}^{2}+2\Delta t\kappa {∥\nabla \vartheta^{k+1}∥}^{2}=2\Delta t\left(\Psi^{k+1},\vartheta^{k+1}\right).$$

For the first equation of (17), taking inner product with $2\Delta t\upsilon^{k+1}$, we achieve

(22) $$\begin{array}{cc} ∥\upsilon^{k+1}{∥}^{2}-∥{\tilde{\upsilon }}_{☆}^{k}{∥}^{2}+{∥\upsilon^{k+1}-{\tilde{\upsilon }}_{☆}^{k}∥}^{2}\; & -2\Delta t(\upsilon^{k+1},R_{e}^{-1}\nabla \times \left(\nabla \times {\tilde{\upsilon }}^{k}\right)+\upsilon^{k}\cdot \nabla {\tilde{\upsilon }}^{k})\\ \; & =2\Delta t(f^{k+1}+\beta \vartheta^{k+1},\upsilon^{k+1}). \end{array}$$

Taking inner product with itself for the first equation of (14), we get

(23) $$∥{\tilde{\upsilon }}^{k+1}{∥}^{2}+\Delta t^{2}∥\alpha^{k+1}{∥}^{2}+2\Delta t\left({\tilde{\upsilon }}^{k+1},\alpha^{k+1}\right)=∥\upsilon^{k+1}{∥}^{2}+\Delta t^{2}{∥\alpha^{k}∥}^{2}+2\Delta t\left(\upsilon^{k+1},\alpha^{k}\right),$$

with the definition of $\xi^{k}$, we obtain

$$\begin{array}{cc} \left({\tilde{\upsilon }}^{k+1},\alpha^{k+1}\right) & =({\tilde{\upsilon }}^{k+1},R_{e}^{-1}\nabla \times \left(\nabla \times {\tilde{\upsilon }}^{k+1}\right)+\upsilon^{k+1}\cdot \nabla {\tilde{\upsilon }}^{k+1}-R_{e}^{-1}\nabla \xi^{k+1})\\ \; & =({\tilde{\upsilon }}^{k+1},R_{e}^{-1}\nabla \times \left(\nabla \times {\tilde{\upsilon }}^{k+1}\right)-R_{e}^{-1}\nabla \xi^{k+1})\\ \; & =R_{e}^{-1}{∥\nabla \times {\tilde{\upsilon }}^{k+1}∥}^{2}-R_{e}^{-1}\left({\tilde{\upsilon }}^{k+1},\nabla \xi^{k+1}\right)\\ \; & =R_{e}^{-1}{∥\nabla \times {\tilde{\upsilon }}^{k+1}∥}^{2}+R_{e}^{-1}\left(\nabla \cdot {\tilde{\upsilon }}^{k+1},\xi^{k+1}\right)\\ \; & =R_{e}^{-1}{∥\nabla \times {\tilde{\upsilon }}^{k+1}∥}^{2}+R_{e}^{-1}\left(\rho^{k+1}-\xi^{k},\xi^{k+1}\right)\\ \; & =R_{e}^{-1}∥\nabla \times {\tilde{\upsilon }}^{k+1}{∥}^{2}+{2R_{e}}^{-1}(∥\xi^{k+1}{∥}^{2}-∥\xi^{k}{∥}^{2}+∥\xi^{k+1}-\xi^{k}{∥}^{2}), \end{array}$$

next, we calculate

$$\begin{array}{cc} \left(\upsilon^{k+1},\alpha^{k}\right) & =(\upsilon^{k+1},R_{e}^{-1}\nabla \times \left(\nabla \times {\tilde{\upsilon }}^{k}\right)+\upsilon^{k}\cdot \nabla {\tilde{\upsilon }}^{k}-R_{e}^{-1}\nabla \rho^{k})\\ \; & =(\upsilon^{k+1},R_{e}^{-1}\nabla \times \left(\nabla \times {\tilde{\upsilon }}^{k}\right)+\upsilon^{k}\cdot \nabla {\tilde{\upsilon }}^{k}), \end{array}$$

with

$$\xi^{k+1}-\xi^{k}=\nabla \cdot {\tilde{\upsilon }}^{k+1},$$

using equation ${∥\nabla \times u∥}^{2}{+∥\nabla \cdot u∥}^{2}={∥\nabla u∥}^{2},\forall u\in X$, we realize

$$2R_{e}^{-1}\Delta t∥\nabla \times {\tilde{\upsilon }}^{k+1}{∥}^{2}+R_{e}^{-1}\Delta t∥\nabla \cdot {\tilde{\upsilon }}^{k+1}{∥}^{2}=R_{e}^{-1}\Delta t∥\nabla \times {\tilde{\upsilon }}^{k+1}{∥}^{2}+R_{e}^{-1}\Delta t{∥\nabla {\tilde{\upsilon }}^{k+1}∥}^{2},$$

so (23) becomes

(24) $$\begin{array}{cc} \; & ∥{\tilde{\upsilon }}^{k+1}{∥}^{2}-∥\upsilon^{k+1}{∥}^{2}+R_{e}^{-1}\Delta t∥\nabla {\tilde{\upsilon }}^{k+1}{∥}^{2}+R_{e}^{-1}\Delta t{∥\nabla \times {\tilde{\upsilon }}^{k+1}∥}^{2}\\ \; & +\Delta t^{2}(∥\alpha^{k+1}{∥}^{2}-∥\alpha^{k}{∥}^{2})+R_{e}^{-1}\Delta t(∥\xi^{k+1}{∥}^{2}-∥\xi^{k}{∥}^{2})\\ \; & +2\Delta t(\upsilon^{k+1},R_{e}^{-1}\nabla \times \left(\nabla \times {\tilde{\upsilon }}^{k}\right)+\upsilon^{k}\cdot \nabla {\tilde{\upsilon }}^{k})=0. \end{array}$$

Finally, summing up (19)–(22), (24) from k=0 to N−1 and getting rid of some positive terms, there holds

(25) $$\begin{array}{cc} \; & S∥b^{N}{∥}^{2}+∥\vartheta^{N}{∥}^{2}+∥{\tilde{\upsilon }}^{N}{∥}^{2}+\Delta t^{2}∥\alpha^{N}{∥}^{2}+R_{e}^{-1}\Delta t∥\xi^{N}{∥}^{2}+2\Delta t\sum_{k=1}^{N}(\kappa ∥\nabla \vartheta^{k}{∥}^{2}\\ \; & +SR_{m}^{-1}∥\nabla \times b^{k}{∥}^{2})+R_{e}^{-1}\Delta t\sum_{k=1}^{N}(∥\nabla {\tilde{\upsilon }}^{k}{∥}^{2}+∥\nabla \times {\tilde{\upsilon }}^{k}{∥}^{2})\leq S∥b^{0}{∥}^{2}+{∥\vartheta^{0}∥}^{2}\\ \; & +∥{\tilde{\upsilon }}^{0}{∥}^{2}+\Delta t^{2}∥\alpha^{0}{∥}^{2}+R_{e}^{-1}\Delta t{∥\xi^{0}∥}^{2}+2\Delta t\sum_{k=1}^{N}\left(\left(f^{k}+\beta \vartheta^{k},\upsilon^{k}\right)+S\left(g^{k},b^{k}\right)+\left(\Psi^{k},\vartheta^{k}\right)\right). \end{array}$$

thus, the proof is ended. □

## 4. Numerical Experiments

In this section, we provide some numerical examples to validate the efficiency and accuracy of the RVC scheme for the time-dependent thermally coupled MHD problem. The first is to prove the optimal convergence performance of the scheme. The second is a thermal driven cavity flow problem without an exact solution. The last is sinusoidal hot cylinder problem. We use uniform finite elements $P_{1}^{b}$ to discrete υ and ϑ, the b is implemented by H(curl)-conforming $N\overset{´}{e}d\overset{´}{e}\mathrm{lec}$ element, the continuous $P_{1}$ finite element for discretizing *p*.

### 4.1. Exact Solution with a Smooth Solution

In the first experiment, we take into account exact solution problem to test the convergence results. A smooth solution is presented in Ω=[0,1]×[0,1], we assume the following functions

$$\left\{\begin{array}{c} \upsilon =(ax_{1}^{2}{\left(x_{1}^{2}-1\right)}^{2}x_{2}\left(x_{2}-1\right)\cos\left(t\right),\;-ax_{2}^{2}{\left(x_{2}^{2}-1\right)}^{2}x_{2}\left(x_{1}-1\right)\cos\left(t\right)),\\ b=(a\sin\left(\pi x_{1}\right)\cos\left(\pi x_{2}\right)\cos\left(t\right),\;-a\sin\left(\pi x_{2}\right)\cos\left(\pi x_{1}\right)\cos\left(t\right)),\\ \vartheta =ax_{1}^{2}{\left(x_{1}^{2}-1\right)}^{2}x_{2}\left(x_{2}-1\right)\cos\left(t\right)-ax_{2}^{2}{\left(x_{2}^{2}-1\right)}^{2}x_{1}\left(x_{1}-1\right)\cos\left(t\right),\\ p=a\left(2x_{1}-1\right)\left(2x_{2}-1\right)\cos\left(t\right). \end{array}\right.$$

We set as *a* = $R_{e}$ = $R_{m}$ = *S* = κ = 1, β = (0,1), the time size $\Delta t=h^{2}$ and the decreasing mesh sizes $\frac{1}{50}$, $\frac{1}{60}$, $\frac{1}{70}$, $\frac{1}{80}$. The numerical error results of each variable is shown in Table 1. We can get that each variable reaches the optimal error; namely, the error order of the $L^{2}$-norm of υ, b and ϑ are $O(h^{2})$, O(h) and $O(h^{2})$, respectively. The $H^{1}$-norm error results of υ and ϑ are O(h), the H(curl)-norm error result of b is O(h). In addition, the $L^{2}$-norm error result of *p* is $O(h^{\frac{3}{2}})$ which is consistent with reference [15]. Since one of the advantages of the RVC scheme overcomes the difficulty caused by the artificial pressure Neumann boundary condition. Then we mainly changed the equation coefficients, namely $R_{e}$, $R_{m}$, κ and *a* to verify that the change of the coefficients will reduce the $L^{2}$ convergence order of the υ, and the convergence order of other variables remains optimal, see Table 2, Table 3, Table 4 and Table 5.

**Figure 2:**
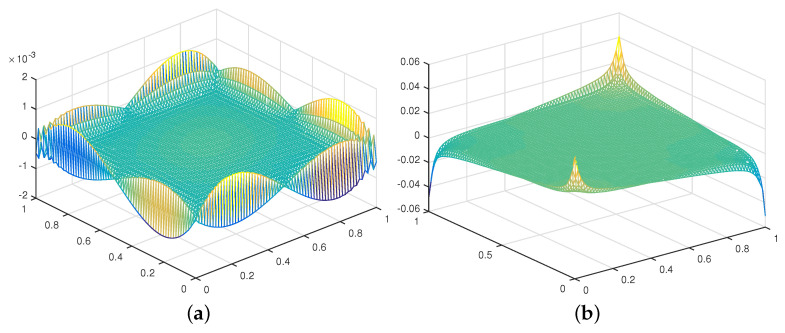
Pressure error at *T* = 1: (**a**) SVC and (**b**) RVC.

In addition, we show the pressure error field at T=1, the time size $\Delta t=h^{2}$, $h=\frac{1}{64}$ for a typical time step using the SVC and the RVC schemes refer to [15]. Figure 2a produces a numerical boundary layer, there is no numerical boundary layer in Figure 2b, but we observe large spikes at the four corners of the domain. This test suggests that the divergence correction of the rotational form successfully cured the numerical boundary layer problem. However, the large errors at the four corners degrade the global convergence rate of the pressure approximation.

**Figure 3:**
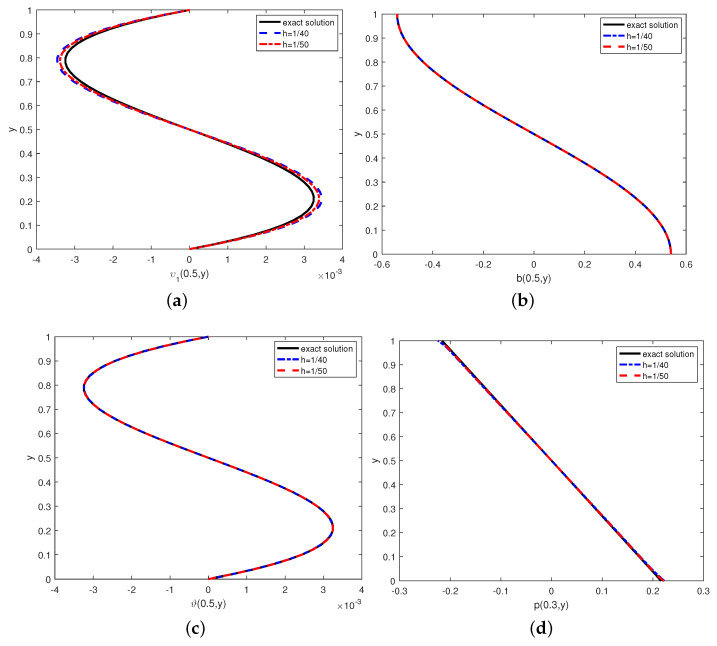
(**a**–**d**) Exact solutions and numerical solutions under the different mesh sizes at *T* = 1.

Finally, we also do the basic agreement between the exact solutions and numerical solutions of the variables at different grid in Figure 3. From this, we find that the coarseness of the grid has little effect on the error.

### 4.2. Thermal Driven Cavity Flow Problem

**Figure 4:**
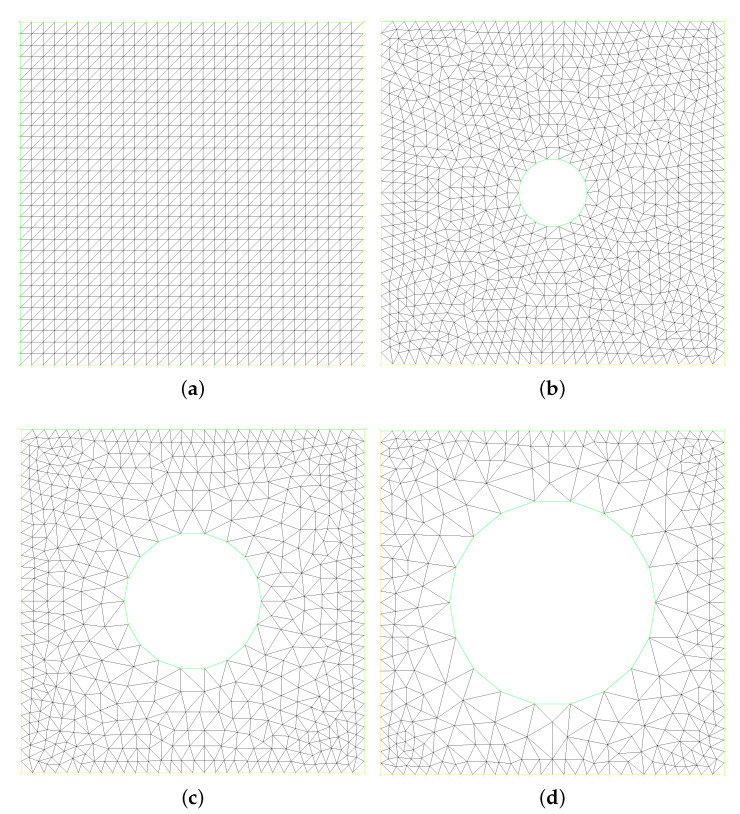
Physics model of structured fine mesh (**a**) and non-structured fine mesh (**b**–**d**).

In the second experiment, we consider the thermal driven flow, which testing the domain Ω=[0,1]×[0,1], the source terms f = g = 0, Ψ=0, the initial values $\upsilon_{0}$ = $b_{0}$ = 0, $\vartheta_{0}$ = 0, the time size Δt=0.001, the mesh sizes $\frac{1}{64}$ in Figure 4a, and following boundary conditions which can be refer to [4,26]:

(26) $$\left\{\begin{array}{c} \upsilon =(0,0),\;\mathrm{on}\;\partial \Omega ,\\ \vartheta =0,\;\mathrm{on}\;x_{1}=1,\\ \vartheta =1,\;\mathrm{on}\;x_{1}=0,\\ n\times b=n\times b_{D},\;\mathrm{on}\;\partial \Omega , \end{array}\right.$$

where $b_{D}=\left(0,1\right)$.

**Figure 5:**
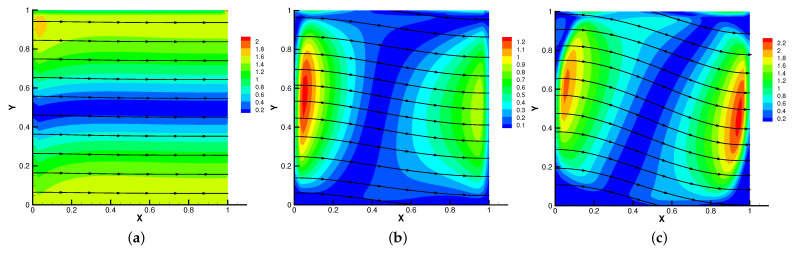
The magnetic field for t=0.1 (**a**), t=0.5 (**b**), t=1 (**c**).

**Figure 6:**
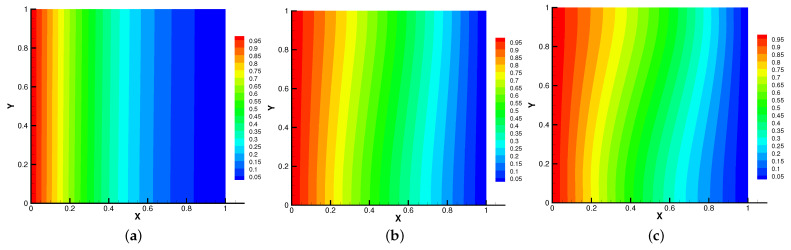
The isotherms for t=0.1 (**a**), t=0.5 (**b**), t=1 (**c**).

**Figure 7:**
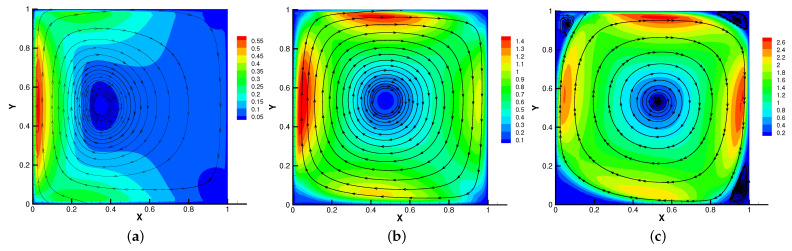
The velocity streamlines for t=0.1 (**a**), t=0.5 (**b**), t=1 (**c**).

**Figure 8:**
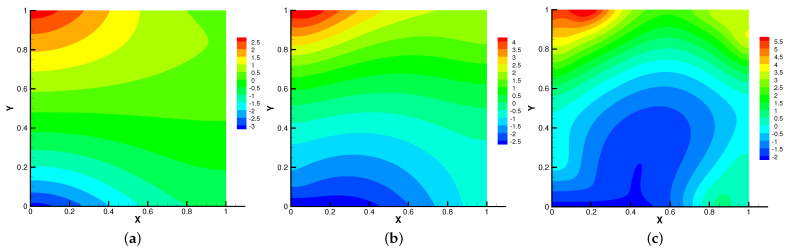
The isobars for t=0.1 (**a**), t=0.5 (**b**), t=1 (**c**).

In Figure 5, Figure 6, Figure 7 and Figure 8, we compare the results of the magnetic, temperature, velocity and pressure fields for T=0.1,0.5,1 when the coefficients are the same. With the change of time, the magnetic field streamlines gradually bend, and the vortex of the velocity streamlines gradually move to the right. There will be three more vortex in the square cavity except the lower left corner, and the temperature and pressure will also produce obvious changes at T=1.

**Figure 9:**
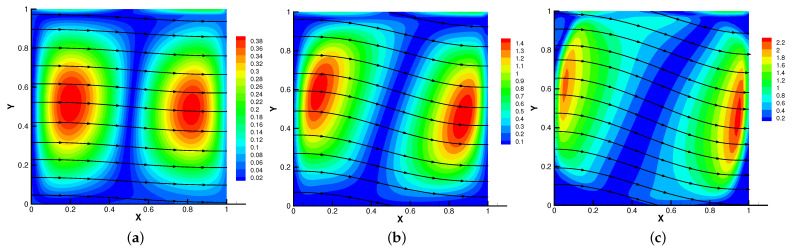
The magnetic field for $R_{e}=10$ (**a**), $R_{e}=100$ (**b**), $R_{e}=1000$ (**c**).

**Figure 10:**
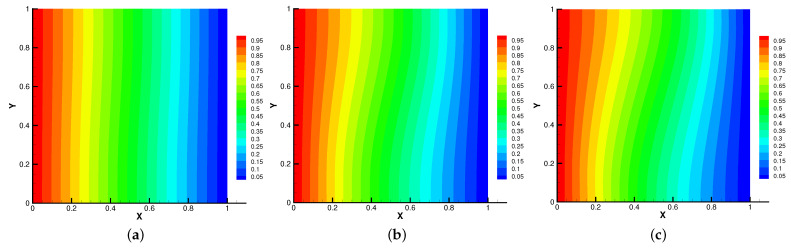
The isotherms for $R_{e}=10$ (**a**), $R_{e}=100$ (**b**), $R_{e}=1000$ (**c**).

**Figure 11:**
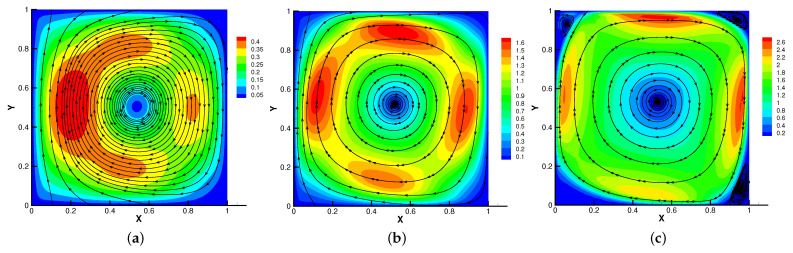
The velocity streamlines for $R_{e}=10$ (**a**), $R_{e}=100$ (**b**), $R_{e}=1000$ (**c**).

**Figure 12:**
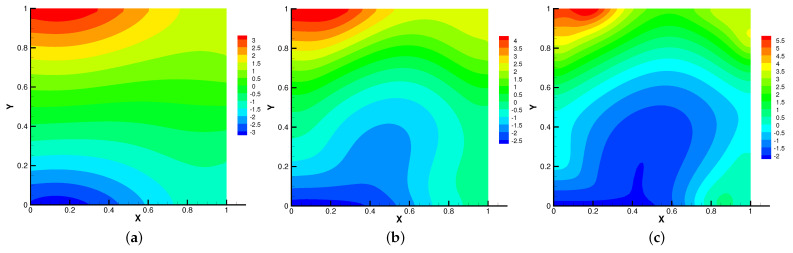
The isobars for $R_{e}=10$ (**a**), $R_{e}=100$ (**b**), $R_{e}=1000$ (**c**).

In Figure 9, Figure 10, Figure 11 and Figure 12, we compare the effect of $R_{e}$ with β = (0,1), *S* = $R_{m}$ = κ = 1. With the continuous increase of $R_{e}$, the magnetic field streamlines have obvious bend, at the same time, the velocity vortex moves to the left and a small vortex is generated at the corner of the area when $R_{e}=1000$. The isotherm is bent because the high temperature liquid flows to the low temperature and the low temperature liquid flows to the high temperature. In Figure 12, the pressure shows a gradual decrease.

### 4.3. Sinusoidal Hot Cylinder Problem

Last, we test an important practical problem, called sinusoidal hot cylinder problem, which has been investigated in [28]. We define the test domain Ω=[0,1]×[0,1], the source terms f = g = 0, Ψ=0, the initial values $\upsilon_{0}$ = $b_{0}$ = 0, $\vartheta_{0}$ = 0, the time sizes Δt=0.000625, the mesh size $\frac{1}{40}$ in Figure 4b–d and the boundary conditions are given as follows:

(27) $$\left\{\begin{array}{c} \upsilon =(0,0),\;\mathrm{on}\;\mathrm{all}\;\mathrm{walls},\\ \frac{\partial \vartheta }{\partial n}=1,\;\mathrm{on}\;\mathrm{inner}\;\mathrm{wall},\\ \vartheta =0,\;\mathrm{on}\;\mathrm{other}\;\mathrm{walls},\\ n\times b=n\times b_{D},\;\mathrm{on}\;\mathrm{all}\;\mathrm{walls}, \end{array}\right.$$

where $b_{D}=\left(0,1\right)$.

**Figure 13:**
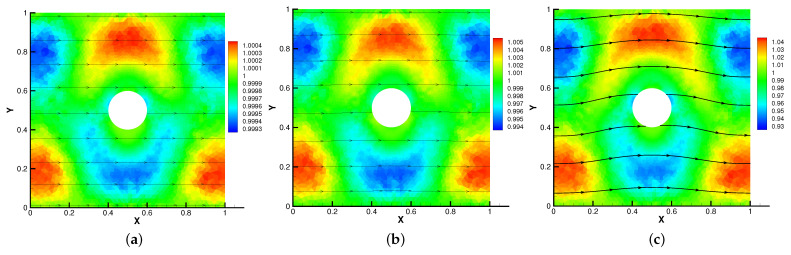
The magnetic field for β=(0,10) (**a**), β=(0,100) (**b**), β=(0,1000) (**c**) with $r_{in}=0.1$.

**Figure 14:**
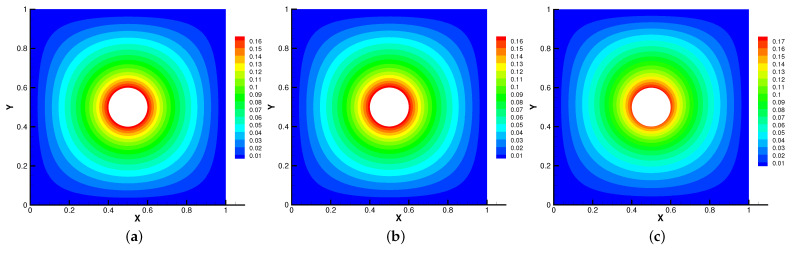
The isotherms for β=(0,10) (**a**), β=(0,100) (**b**), β=(0,1000) (**c**) with $r_{in}=0.1$.

**Figure 15:**
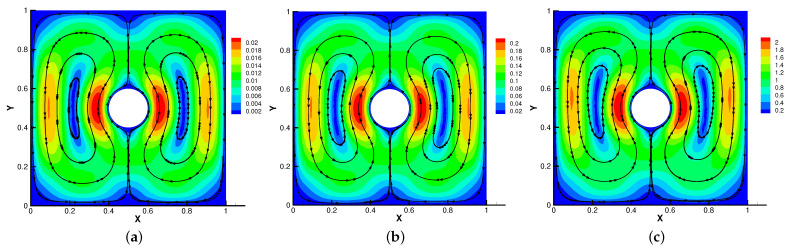
The velocity streamlines for β=(0,10) (**a**), β=(0,100) (**b**), β=(0,1000) (**c**) with $r_{in}=0.1$.

**Figure 16:**
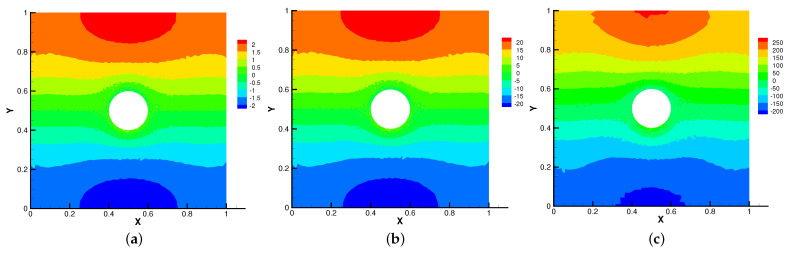
The isobars for β=(0,10) (**a**), β=(0,100) (**b**), β=(0,1000) (**c**) with $r_{in}=0.1$.

Firstly, we set the radius of inner cylinder $\left(r_{in}=0.1\right)$, $R_{e}$ = *S* = $R_{m}$ = κ = 1 for different β=(0,10),(0,100),(0,1000). In Figure 13, Figure 14, Figure 15 and Figure 16, we find that the magnetic field lines are slightly curved. The high temperature circle gradually shifts upward in the center of the Figure 14. Two main vortices are captured in velocity filed, along with β increases the velocity streamlines follow the shape of a cylinder, the two main vortices, it is obvious that the vortex in the center has changed from slender to stubby. In Figure 16, the pressure difference is decreasing as the fluid flows.

**Figure 17:**
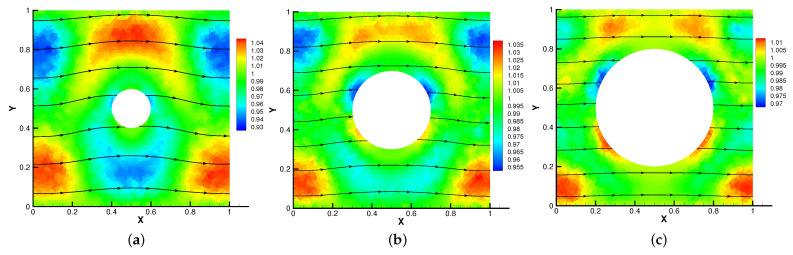
The magnetic field for $r_{in}=0.1$ (**a**), $r_{in}=0.2$ (**b**), $r_{in}=0.3$ (**c**) with β=(0,1000).

**Figure 18:**
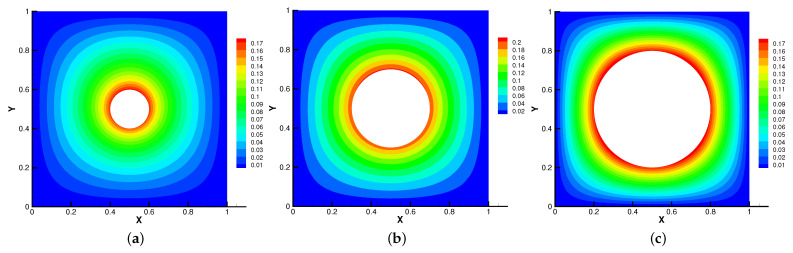
The isotherms for $r_{in}=0.1$ (**a**), $r_{in}=0.2$ (**b**), $r_{in}=0.3$ (**c**) with β=(0,1000).

**Figure 19:**
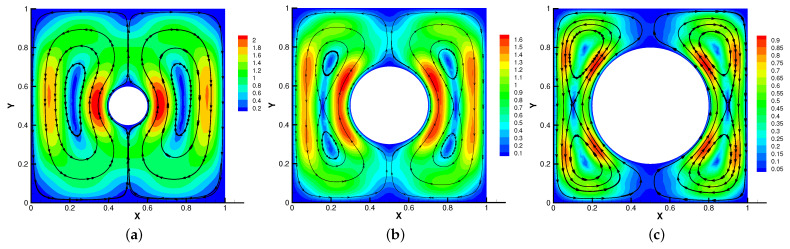
The velocity streamlines for $r_{in}=0.1$ (**a**), $r_{in}=0.2$ (**b**), $r_{in}=0.3$ (**c**) with β=(0,1000).

**Figure 20:**
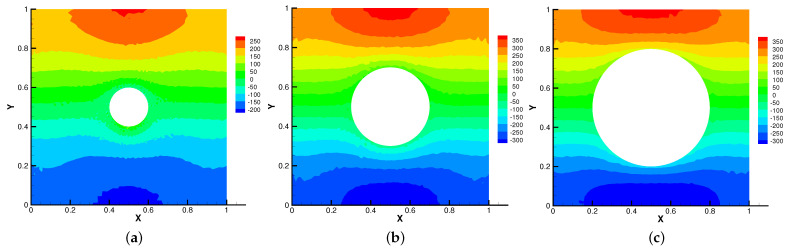
The isobars for $r_{in}=0.1$ (**a**), $r_{in}=0.2$ (**b**), $r_{in}=0.3$ (**c**) with β=(0,1000).

Then, we set β=(0,1000) and $R_{e}$ = *S* = $R_{m}$ = κ = 1 for different radius of inner cylinder $\left(r_{in}=0.1,0.2,0.3\right)$ in Figure 17, Figure 18, Figure 19 and Figure 20. As the radius of the inner cylinder gradually increases, the streamlines of the magnetic field gradually flatten from bending, the fluid does not have enough space for rotation, so the conduction mode dominates. Besides, four vortices are captured. The isotherm follows the shape of the cylinder, and the upward movement of the two main vortices gradually disappears. The pressure changes obviously with the increasing of the radius of the cylinder.

The above phenomena found in Figure 13, Figure 14, Figure 15, Figure 16, Figure 17, Figure 18, Figure 19 and Figure 20 are very consistent with [28,29,30]. Therefore, Our method can simulate this model well.

## 5. Conclusions

In this paper, we accomplished the following goals: (i) give a linear full decoupling velocity correction schemes for thermally coupled incompressible MHD system; (ii) the stability of the proposed algorithms are derived; (iii) the numerical results verified the reliability and accuracy of the proposed RVC algorithm. The above numerical experiments show that the RVC method is a powerful tool for solving the problem and can deal with complex problem. In the later work, we will go on study about full decoupling numerical method for settling unsteady thermally coupled incompressible MHD equations. 

## Figures and Tables

**Table 1:** The convergence rates for heat-MHD equations with $a=R_{e}=R_{m}=\alpha =\kappa =S=1$.

h	$∥\upsilon -\upsilon_{h}∥$	Ratio	${\left|\upsilon -\upsilon \right|}_{1}$	Ratio	$∥b-b_{h}∥$	Ratio	$|b-b_{h}{|}_{1}$	Ratio
1/40	2.06 × $10^{-5}$		1.87 × $10^{-3}$		1.60 × $10^{-2}$		8.34 × $10^{-2}$	
1/50	1.31 × $10^{-5}$	2.02	1.49 × $10^{-3}$	1.01	1.28 × $10^{-2}$	1.00	6.67 × $10^{-2}$	1.00
1/60	9.09 × $10^{-6}$	2.02	1.24 × $10^{-3}$	1.00	1.06 × $10^{-2}$	1.00	5.56 × $10^{-2}$	1.00
1/70	6.66 × $10^{-6}$	2.01	1.06 × $10^{-3}$	1.00	9.12 × $10^{-3}$	1.00	4.76 × $10^{-2}$	1.00
1/80	5.09 × $10^{-6}$	2.01	9.32 × $10^{-4}$	1.00	7.98 × $10^{-3}$	1.00	4.17 × $10^{-2}$	1.00
**h**	$∥\vartheta -\vartheta_{h}∥$	**Ratio**	$|\vartheta -\vartheta_{h}{|}_{1}$	**Ratio**	$∥p-p_{h}∥$	**Ratio**		
1/40	9.61 × $10^{-6}$		1.37 × $10^{-3}$		2.96 × $10^{-4}$			
1/50	6.15 × $10^{-6}$	2.00	1.10 × $10^{-3}$	1.00	2.01 × $10^{-4}$	1.73		
1/60	4.27 × $10^{-6}$	2.00	9.15 × $10^{-4}$	1.00	1.47 × $10^{-4}$	1.71		
1/70	3.14 × $10^{-6}$	2.00	7.84 × $10^{-4}$	1.00	1.14 × $10^{-4}$	1.69		
1/80	2.40 × $10^{-6}$	2.00	6.86 × $10^{-4}$	1.00	9.08 × $10^{-5}$	1.68		

**Table 2:** The convergence rates for heat-MHD equations with $R_{e}=0.1,$$R_{m}=0.1$ and a=κ=S=1.

h	$∥\upsilon -\upsilon_{h}∥$	Ratio	${\left|\upsilon -\upsilon \right|}_{1}$	Ratio	$∥b-b_{h}∥$	Ratio	$|b-b_{h}{|}_{1}$	Ratio
1/30	2.91 × $10^{-4}$		3.66 × $10^{-3}$		2.13 × $10^{-2}$		1.11 × $10^{-1}$	
1/40	2.14 × $10^{-4}$	1.07	2.82 × $10^{-3}$	0.90	1.60 × $10^{-2}$	1.00	8.34 × $10^{-2}$	1.00
1/50	1.62 × $10^{-4}$	1.26	2.27 × $10^{-3}$	0.98	1.28 × $10^{-2}$	1.00	6.67 × $10^{-2}$	1.00
1/60	1.26 × $10^{-4}$	1.38	1.88 × $10^{-3}$	1.01	1.06 × $10^{-2}$	1.00	5.56 × $10^{-2}$	1.00
**h**	$∥\vartheta -\vartheta_{h}∥$	**Ratio**	$|\vartheta -\vartheta_{h}{|}_{1}$	**Ratio**	$∥p-p_{h}∥$	**Ratio**		
1/30	1.70 × $10^{-5}$		1.83 × $10^{-3}$		3.25 × $10^{-2}$			
1/40	9.58 × $10^{-6}$	2.00	1.37 × $10^{-3}$	1.00	2.44 × $10^{-2}$	1.00		
1/50	6.13 × $10^{-6}$	2.00	1.10 × $10^{-3}$	1.00	1.91 × $10^{-2}$	1.11		
1/60	4.23 × $10^{-6}$	2.00	9.15 × $10^{-4}$	1.00	1.53 × $10^{-2}$	1.19		

**Table 3:** The convergence rates for heat-MHD equations with $R_{e}=2.5,$$R_{m}=2.5$ and a=κ=S=1.

h	$∥\upsilon -\upsilon_{h}∥$	Ratio	${\left|\upsilon -\upsilon \right|}_{1}$	Ratio	$∥b-b_{h}∥$	Ratio	$|b-b_{h}{|}_{1}$	Ratio
1/30	5.81 × $10^{-5}$		2.65 × $10^{-3}$		2.13 × $10^{-2}$		1.11 × $10^{-1}$	
1/40	3.39 × $10^{-5}$	1.87	1.95 × $10^{-3}$	1.07	1.60 × $10^{-2}$	1.00	8.34 × $10^{-2}$	1.00
1/50	2.22 × $10^{-5}$	1.90	1.54 × $10^{-3}$	1.05	1.28 × $10^{-2}$	1.00	6.67 × $10^{-2}$	1.00
1/60	1.56 × $10^{-5}$	1.92	1.28 × $10^{-3}$	1.04	1.06 × $10^{-2}$	1.00	5.56 × $10^{-2}$	1.00
**h**	$∥\vartheta -\vartheta_{h}∥$	**Ratio**	$|\vartheta -\vartheta_{h}{|}_{1}$	**Ratio**	$∥p-p_{h}∥$	**Ratio**		
1/30	1.70 × $10^{-5}$		1.83 × $10^{-3}$		4.72 × $10^{-4}$			
1/40	9.58 × $10^{-6}$	2.00	1.37 × $10^{-3}$	1.00	2.92 × $10^{-4}$	1.67		
1/50	6.13 × $10^{-6}$	2.00	1.10 × $10^{-3}$	1.00	2.02 × $10^{-4}$	1.65		
1/60	4.23 × $10^{-6}$	2.00	9.15 × $10^{-4}$	1.00	1.50 × $10^{-4}$	1.63		

**Table 4:** The convergence rates for heat-MHD equations with $R_{e}=R_{m}=0.1$ and a=0.1, κ=S=1.

h	$∥\upsilon -\upsilon_{h}∥$	Ratio	${\left|\upsilon -\upsilon \right|}_{1}$	Ratio	$∥b-b_{h}∥$	Ratio	$|b-b_{h}{|}_{1}$	Ratio
1/30	1.72 × $10^{-4}$		9.11 × $10^{-4}$		2.13 × $10^{-2}$		1.11 × $10^{-1}$	
1/40	1.08 × $10^{-4}$	1.87	6.18 × $10^{-4}$	1.07	1.60 × $10^{-2}$	1.00	8.34 × $10^{-2}$	1.00
1/50	7.35 × $10^{-5}$	1.90	4.54 × $10^{-4}$	1.05	1.28 × $10^{-2}$	1.00	6.67 × $10^{-2}$	1.00
1/60	5.31 × $10^{-5}$	1.92	3.51 × $10^{-4}$	1.04	1.06 × $10^{-2}$	1.00	5.56 × $10^{-2}$	1.00
**h**	$∥\vartheta -\vartheta_{h}∥$	**Ratio**	$|\vartheta -\vartheta_{h}{|}_{1}$	**Ratio**	$∥p-p_{h}∥$	**Ratio**		
1/30	1.71 × $10^{-6}$		1.83 × $10^{-3}$		4.49 × $10^{-2}$			
1/40	9.61 × $10^{-7}$	2.00	1.37 × $10^{-3}$	1.00	4.94 × $10^{-2}$	1.67		
1/50	6.15 × $10^{-7}$	2.00	1.10 × $10^{-3}$	1.00	5.19 × $10^{-2}$	1.65		
1/60	4.23 × $10^{-7}$	2.00	9.15 × $10^{-4}$	1.00	5.34 × $10^{-2}$	1.63		

**Table 5:** The convergence rates for heat-MHD equations with κ=0.1 and $a=R_{e}=R_{m}=S=1$.

h	$∥\upsilon -\upsilon_{h}∥$	Ratio	${\left|\upsilon -\upsilon \right|}_{1}$	Ratio	$∥b-b_{h}∥$	Ratio	$|b-b_{h}{|}_{1}$	Ratio
1/30	3.69 × $10^{-5}$		2.50 × $10^{-3}$		2.13 × $10^{-2}$		1.11 × $10^{-1}$	
1/40	2.06 × $10^{-5}$	2.02	1.87 × $10^{-3}$	1.01	1.60 × $10^{-2}$	1.00	8.34 × $10^{-2}$	1.00
1/50	1.31 × $10^{-5}$	2.02	1.49 × $10^{-3}$	1.01	1.28 × $10^{-2}$	1.00	6.67 × $10^{-2}$	1.00
1/60	9.08 × $10^{-6}$	2.02	1.24 × $10^{-3}$	1.00	1.06 × $10^{-2}$	1.00	5.56 × $10^{-2}$	1.00
**h**	$∥\vartheta -\vartheta_{h}∥$	**Ratio**	$|\vartheta -\vartheta_{h}{|}_{1}$	**Ratio**	$∥p-p_{h}∥$	**Ratio**		
1/30	1.47 × $10^{-5}$		1.83 × $10^{-3}$		4.91 × $10^{-4}$			
1/40	8.26 × $10^{-6}$	2.00	1.37 × $10^{-3}$	1.00	2.96 × $10^{-4}$	1.76		
1/50	5.29 × $10^{-6}$	2.00	1.10 × $10^{-3}$	1.00	2.01 × $10^{-4}$	1.73		
1/60	3.67 × $10^{-6}$	2.00	9.15 × $10^{-4}$	1.00	1.47 × $10^{-4}$	1.71		

## Data Availability

Not applicable.

## Abbreviations

**List of Abbreviated Symbols**	
MHD	magneto-hydrodynamic
SVC	standard velocity correction
RVC	rotation velocity correction
**Physical parameters**	
α:=LU	Thermal diffusivity
$R_{e}:=\frac{\rho UL}{\eta }$	Reynolds number
$S:=\frac{B}{\mu \rho U^{2}}$	Coupling number
$\beta :=\frac{\rho g\delta \vartheta L^{3}}{\alpha \eta }$	Thermal expansion coefficient
$R_{m}:=\mu \sigma LU$	Magnetic Reynolds number
**Greek symbols**	
*L*	characteristic length
*U*	characteristic velocity
*B*	characteristic magnetic field
g	gravitational acceleration vector
δϑ	characteristic temperature difference
ρ	fluid density
μ	dynamic viscosity
η	electrical conductance
σ	magnetic diffusivity
